# Adaptogenic‐Like Potential of *Bauhinia forficata* Leaf Extracts: Behavioral and Oxidative Outcomes in an Acute Stress Mouse Model

**DOI:** 10.1002/brb3.71819

**Published:** 2026-10-08

**Authors:** Sofia Alencar Lafetá, Thiago Neves Vieira, Rodrigo Rodrigues Franco, Ana Claudia Gontijo Couto, Vinicius Prado Bittar, Maria Sol Peña Carrillo, Renata Roland Teixeira, André Luis Lopes Saraiva, Allisson Benatti Justino, Tamara Silva, Veridiana de Melo Rodrigues Ávila, Foued Salmen Espíndola, Cássia Regina Silva

**Affiliations:** ^1^ Analgesia and Inflammation Research Group–GPANI; Laboratory of Biochemistry and Animal Toxins–LABITOX, Institute of Biotechnology Federal University of Uberlândia Uberlândia Minas Gerais Brazil; ^2^ Laboratory of Biochemistry and Molecular Biology–LABIBI, Institute of Biotechnology Federal University of Uberlândia Uberlândia Minas Gerais Brazil; ^3^ Department of Medicine, Institute of Biotechnology Federal University of Catalão Catalão Goiás Brazil

**Keywords:** adaptogens, anxiety, depression, natural products, oxidative stress

## Abstract

**Introduction:**

Chronic stress exacerbates anxiety and depression, prompting interest in adaptogens that enhance resilience via redox modulation. Building on its established systemic antioxidant properties and traditional use as a tonic, we hypothesized that flavonoid‐rich *Bauhinia forficata* leaves possess unexplored adaptogenic‐like potential in the central nervous system. Therefore, this study aimed to assess the adaptogenic‐like potential of crude extracts (CEs) and dichloromethane (DCM) fraction from *B. forficata leaves* in an acute stress mouse model.

**Methods:**

Adult male C57BL/6J/UFU mice were divided into two independent experimental sets. The first set evaluated the CE (five groups [*n* = 8–9 per group]: naive, stress plus vehicle, and stress plus CE at 30, 100, or 300 mg/kg). The second set evaluated the DCM fraction (five groups [*n* = 8–9 per group]: naive, stress plus vehicle, and stress plus DCM fraction at 10, 30, or 100 mg/kg). Treatments were administered orally for 4 days, beginning 2 days before stress induction. A 12‐h social isolation protocol without food or water induced acute stress. Behavioral assessments included the forced swim test (depressive‐like behavior), elevated plus maze (anxiety‐like behavior), and open field test (anxiety and locomotor activity). Serum urea and alanine aminotransferase (ALT) levels assessed potential hepatic and renal damage. Redox biomarkers such as superoxide dismutase, catalase, glutathione peroxidase, glutathione reductase, glutathione content, ferric reducing antioxidant power, sulfhydryl groups, reactive oxygen species, and lipid peroxidation were quantified in the cortex, striatum, cerebellum, and hippocampus of four groups of mice (*n* = 8 per group): naive, stress plus vehicle, and stress plus CE 30 mg/kg or DCM fraction at 10 mg/kg, respectively.

**Results:**

Both CE (30 mg/kg) and DCM (10 mg/kg) significantly reduced immobility in the forced swim test and increased time spent in the open arms of the elevated plus maze, indicating antidepressant‐ and anxiolytic‐like effects. Higher doses displayed reduced efficacy. No significant differences in serum urea or ALT were detected. Redox assessments revealed that CE and DCM treatments restored antioxidant capacity and decreased lipid peroxidation, particularly in the striatum and cortex. CE and DCM from *B. forficata* leaves exhibit adaptogenic‐like properties, enhancing behavioral resilience and modulating oxidative stress in an acute stress mouse model.

**Conclusion:**

These findings highlight their preventive potential against acute stress‐induced behavioral and oxidative changes, though further mechanistic and translational studies are needed to elucidate molecular targets and clinical applicability.

## Graphical abstract

Graphical abstract ilustrates the adaptogenic‐like potential of crude extracts (CEs) and dichloromethane (DCM) fraction from B. forficata leaves in an acute stress mouse model. C57BL/6J/UFU mice were submitted to a social isolation protocol and both CE (30 mg/kg) and DCM (10 mg/kg) significantly reduced immobility in the forced swim test and increased time spent in the open arms of the elevated plus maze, indicating antidepressant‐ and anxiolytic‐like effects. Treatments also restored antioxidant capacity and decreased lipid peroxidation, particularly in the striatum and cortex.

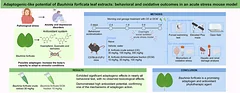

## 1 Introduction

Pathological stress is increasingly prevalent in modern society due to compounding social, economic, and health challenges, including the recent pandemic (Cannizzaro et al. 2019; Manchia et al. 2022). Epidemiological, preclinical, and clinical studies consistently show that chronic or repeated acute stress contributes to the onset and worsening of anxiety and depression. Although conventional pharmacological treatments are available, their utility is often constrained by adverse effects, dependence liability, and delayed onset of action (Garakani et al. 2020). Together, these limitations underscore a need for preventive, well‐tolerated interventions capable of reducing the impact of acute stressors in daily life, such as examinations, public presentations, and job interviews (Anghelescu et al. 2018; Charlson et al. 2019). In this context, natural adaptogens are emerging as promising non‐pharmacological interventions for stress‐related disorders.

Classically, a plant‐derived compound must meet specific pharmacological criteria to be considered an adaptogen: it must have a safe profile (virtually non‐toxic), increase non‐specific resistance to diverse stressors, and exert a normalizing influence on physiological functions (Panossian 2017). At the molecular level, adaptogens achieve this normalization and enhance stress resilience through multi‐system modulation of neuroendocrine, immune, and antioxidant pathways. Their key mechanisms involve HPA axis regulation, oxidative stress reduction, and neurotransmitter rebalancing (Dobrek Ł 2019; Franco et al. 2021; Panossian et al. 2018). These pathways are directly linked to behavioral outcomes. Chronic HPA overactivation and oxidative toxicity act synergistically to induce neuroinflammation and synaptic dysfunction, the structural drivers of depressive‐ and anxiety‐like stress responses (Correia et al. 2023; Hassamal 2023; Lei et al. 2025). As metabolic regulators, adaptogens interrupt this cascade and prevent the morphological damage underlying behavioral deficits. They enhance physiological adaptability by simultaneously driving neurotrophic signaling, modulating cellular stress‐response pathways to bolster endogenous antioxidant defenses, and resolving redox imbalances (Emudainohwo et al. 2023; Franco et al. 2021; Panossian 2017).

Different plant species have been considered as strong candidates for adaptogenic effects because their capacity to correct redox imbalances is intrinsically linked to the normalization of broader neuroendocrine and immune networks (Özdemir and Demir 2025). Adaptogenic botanical extracts are proposed to support resilience by modulating redox homeostasis and attenuating oxidative stress in the central nervous system, thereby helping preserve stress‐response signaling and neuronal function under adverse conditions (Panossian et al. 2020). Within this context, the plant *Bauhinia forficata* (Fabaceae), popularly known as “pata‐de‐vaca,” stands out (Filho 2009; Ferreres et al. 2012; Gudavalli et al. 2024a; Pepato et al. 2004). Native to Brazil, its leaves are traditionally used in teas for their hypoglycemic, tonic, and cardiovascular protective effects (Cechinel‐Zanchett et al. 2019; Córdova Mariángel et al. 2019; Salgueiro et al. 2016; Tonelli et al. 2022). Because chronic stress profoundly disrupts metabolic homeostasis, these traditional metabolic applications provide a conceptual basis to investigate whether *B. forficata* might also exhibit a broader adaptogenic‐like potential to counteract stress‐induced physiological dysfunctions (Franco et al. 2018). Literature reinforces that *B. forficata* prominent antioxidant activity is due to the abundance of phenolic compounds (Filho 2009; Pinafo et al. 2019). More specifically, antioxidant properties of leaves should arise from their ability to eliminate reactive oxygen species (ROS), which are assigned to high amounts of polyphenols such as flavonoids, mainly derivatives of kaempferitrin, quercetin and kaempferol (Filho 2009; Pinafo et al. 2019; Pinheiro et al. 2006; Salgueiro et al. 2016; de Sousa Lino et al. 2004). Beyond general antioxidation, these specific compounds play a critical role in stress neurobiology by modulating glucocorticoid receptor sensitivity. Such modulation promotes neurotrophic signaling pathways, which are essential for maintaining synaptic integrity and neuronal viability during prolonged HPA axis activation (Correia et al. 2023; Lei et al. 2025).

Although adaptogenic compounds are mostly terpenes, phenolic compounds and alkaloids with adaptogenic properties are also known (Franco et al. 2021; Todorova et al. 2021). Flavonoids were previously identified from *B. forficata* leaves mostly in DCM, ethyl acetate and n‐butanol fractions. The DCM fraction was selected for neurobehavioral evaluation due to its high antioxidant potency and lipophilic nature. Furthermore, our group previously demonstrated ability to suppress cellular ROS production in stimulated murine macrophages without inducing cytotoxicity (Franco et al. 2020). Despite these activities, no studies have addressed the plant's adaptogenic potential against acute behavioral symptoms like anxiety and depression. Additionally, a recent clinical trial suggested that the use of capsules containing standardized extract of *B. forficata* can be an adjuvant in diabetic glycemic control. This finding, alongside two other clinical trials using *Bauhinia* tea infusions, supports its safe use in humans and its capacity to regulate metabolic pathways. While these trials focused primarily on glycemic control, the physiological overlap between metabolic dysregulation and chronic stress raises the hypothesis that its systemic effects could extend to stress resilience paradigms (Filho 2009; de Souza et al. 2018; Tonelli et al. 2022).

While direct evidence linking *B. forficata* to neurobehavioral stress resilience remains limited, the established mechanistic overlap between systemic metabolic disruption, cellular oxidative damage, and stress‐induced behavioral dysfunctions provides a strong indirect rationale. Therefore, we hypothesized that the potent redox regulation and cytoprotection previously demonstrated by *B. forficata* would translate into neurobehavioral resilience in an in vivo model. Thus, the objective of this study was to investigate the potential adaptogenic‐like effects of the crude extract (CE) and dichloromethane (DCM) fraction of *B. forficata* leaves by integrating neurobehavioral evaluations of anxiety and depression with site‐specific biochemical analysis of brain redox homeostasis in an acute stress model. By doing so, this study provides a comprehensive assessment of the plant's adaptogenic‐like potential. The findings highlight the investigation of natural products with antioxidant capacity as preventive strategies against acute stress‐induced pathological conditions.

## 2 Materials and Methods

### 2.1 Plant Material and Preparation of Crude Extract and Dichloromethane Fraction

Leaves of adult *B. forficata* subsp. *forficata* plants were harvested in October 2018 (mid‐spring in the Southern Hemisphere) from an urban area within the municipality of Araxá, Minas Gerais, Brazil. The collection site lies in the Cerrado biome at coordinates 19°36′36″ S, 46°57′00″ W, within Brazil's Southeast Region. Immediately after harvesting, the plant material was transported by automobile approximately 180 km to the city of Uberlândia. Upon arrival, the leaves were promptly rinsed to remove debris and then air‐dried in the shade to prevent photodegradation. Drying conditions minimized exposure to insects and dust, ensuring the material's integrity for subsequent extraction. Additionally, a flowering specimen collected concurrently was pressed and deposited as HUFU77094 in the Herbarium of the Federal University of Uberlândia. The dried leaves were subjected to static maceration for 6 days, using a 1:5 (m/v) ratio of plant material to methanol (99.8%). After extraction, the solutions were filtered, and the solvent was removed under reduced pressure at 40°C using a rotary evaporator. This maceration procedure was repeated three times to maximize yield. The combined extracts were subsequently frozen and lyophilized. The CE (30 g) was dissolved in 200 mL of 90% aqueous methanol. This solution underwent liquid‐liquid partitioning using solvents of increasing polarity (hexane, DCM, ethyl acetate, n‐butanol; 200 mL each). The DCM fraction was selected for study due to its efficacy in concentrating terpenes and flavonoids, predominant adaptogenic compounds (Pandey and Tripathi 2014). These fractions were concentrated by rotary evaporation (40°C, reduced pressure), frozen, lyophilized to remove residual water, and stored at −20°C. Subsequent analyses utilized both the crude methanolic extract (CE) and the DCM fraction.

### 2.2 Liquid Chromatography‐Mass Spectrometry Analysis

The compounds present in the DCM fractions were identified by high‐performance liquid chromatography coupled to high‐resolution mass spectrometry with an electrospray ionization source (HPLC‐ESI‐MS/MS, Agilent 6520 Q‐TOF). Chromatographic separation was performed on an Agilent Infinity 1260 liquid chromatograph using a reverse‐phase C18 analytical column (2.1 mm × 50 mm, 1.8 µm particle size). The mobile phases consisted of water acidified with 0.1% (v/v) formic acid (eluent A) and methanol (eluent B). The gradient program was as follows: 10% B at 0 min, ramping to 98% B from 0 to 10 min, held at 98% B for 7 min (10–17 min), followed by re‐equilibration to initial conditions. The flow rate was 0.2 mL/min, and the injection volume was 5 µL. Mass spectrometric detection was carried out in negative and positive electrospray ionization (ESI‐ and ESI+, respectively) modes under the following conditions: nebulizer pressure of 20 psi, drying gas at 8 L/min, drying temperature at 220°C, and capillary voltage set to 4.5 kV. Data acquisition was performed in full‐scan mode across the *m/z* range 100–1200. Tandem mass spectrum (MS/MS) were obtained by collision‐induced dissociation (CID), applying collision energies between 5 and 30 eV to optimize fragmentation patterns for individual compounds. Spectral data were processed using Agilent MassHunter B.07.00 software. The molecular ions and fragmentation profiles were compared with public spectral libraries (MassBank) and previously published literature. Tentative identifications were assigned based on accurate mass measurements and MS/MS fragmentation patterns, using a mass error threshold of ≤ 10 ppm between theoretical and observed *m/z* values. However, it should be noted that compound identifications remain putative, since authentic standards were not available for confirmation. All analyses were performed in triplicate, and results are presented as mean values with standard deviations (see Table 1).

**TABLE 1:** Tentatively identified compounds in the dichloromethane (DCM) fraction of *Bauhinia forficata* subsp. *forficata* leaves, analyzed by HPLC‐ESI‐QTOF‐MS/MS (Agilent 6520) under both positive and negative ionization modes, with collision energies of 5, 10, 15, 20, 25, and 30 eV. Data are presented as mean ± standard deviation (*n* = 3) from independent injection.

Suggested Compounds	Retention time (min)	Molecular Formula	Exact molecular mass	*m/z* of [M+H]^+^	*m/z* of [M‐H]^−^	Calculated mass	*m/z* fragments	Error (ppm)	Reference
Rutin	6.188	C_27_H_30_O_16_	610.1534	—	609.1474	609.1478	10eV: 300	0.65	https://doi.org/10.3390/molecules27175415
Luteolin	11.083	C_15_H_10_O_6_	286.0477	—	285.2086	285.2085	10eV:133	0.35	https://doi.org/10.1016/j.foodchem.2016.05.052
Caffeic acid	4.207	C_9_H_8_O_4_	180.0423	—	179.0340	179.0347	30eV: 135	3.90	https://doi.org/10.1039/C6AY02937J
Ferulic acid	5.282	C_10_H_10_O_4_	194.0579	—	193.0508	193.0511	15eV: 149	1.55	https://doi.org/10.4314/bajopas.v11i1.68S
Kaempferol‐3‐O‐rhamnoside	7.261	C_21_H_20_O_10_	432.1056	—	431.0985	431.0987	15eV: 285	0.46	https://doi.org/10.1016/j.foodchem.2012.02.201
Kaempferol‐3‐O‐rutinoside	6.741	C_27_H_30_O_15_	594.1585	—	593.1491	593.1485	10eV: 285	1.01	https://doi.org/10.1016/j.foodchem.2012.02.201
Kaempferol‐3‐O‐(2‐rhamnosyl) rutinoside	5.819	C_33_H_40_O_19_	740.2164	—	739.2085	739.2087	15eV: 575	0.27	https://doi.org/10.1016/j.foodchem.2012.02.201
Quercetin‐3‐O‐(2‐rhamnosyl) rutinoside	5.416	C_33_H_40_O_20_	756.2113	—	755.2036	755.2047	15eV: 300	1.45	https://doi.org/10.1080/14786419.2018.1460826
Protocatechuic acid	2.412	C_7_H_6_O_4_	154.0266	—	153.0536	153.0545	20eV: 109	5.88	https://doi.org/10.1093/chromsci/bmv240
Lupeol	7.946	C_30_H_50_O	426,3862	426.2870	—	426.2876	15eV: 189	1.40	https://doi.org/10.1111/nph.16013

### 2.3 Animals, Groups, and Treatments

A total of 134 adult male C57BL6/J/UFU mice were kept under standard conditions (22 ± 1°C, humidity 60 ± 5%, and 12 h light/12 h dark) and given nesting conditions, food, and water ad libitum. All animal handling and procedures were approved by the Ethics Committee in Animal Experimentation of the Federal University of Uberlandia (CEUA/UFU 012/20). The overall distribution of the animals was structured as follows: 90 mice were allocated to the behavioral evaluations (10 groups of *n* = 9 divided in two sets); 32 mice were independently assigned to the ex vivo oxidative stress assays (four groups of *n* = 8); and 12 mice were utilized for the biochemical evaluation of hepatic and renal damage (three groups of *n* = 4). The experimental design is illustrated in Figure 1 and structured as follows:

**FIGURE 1:**
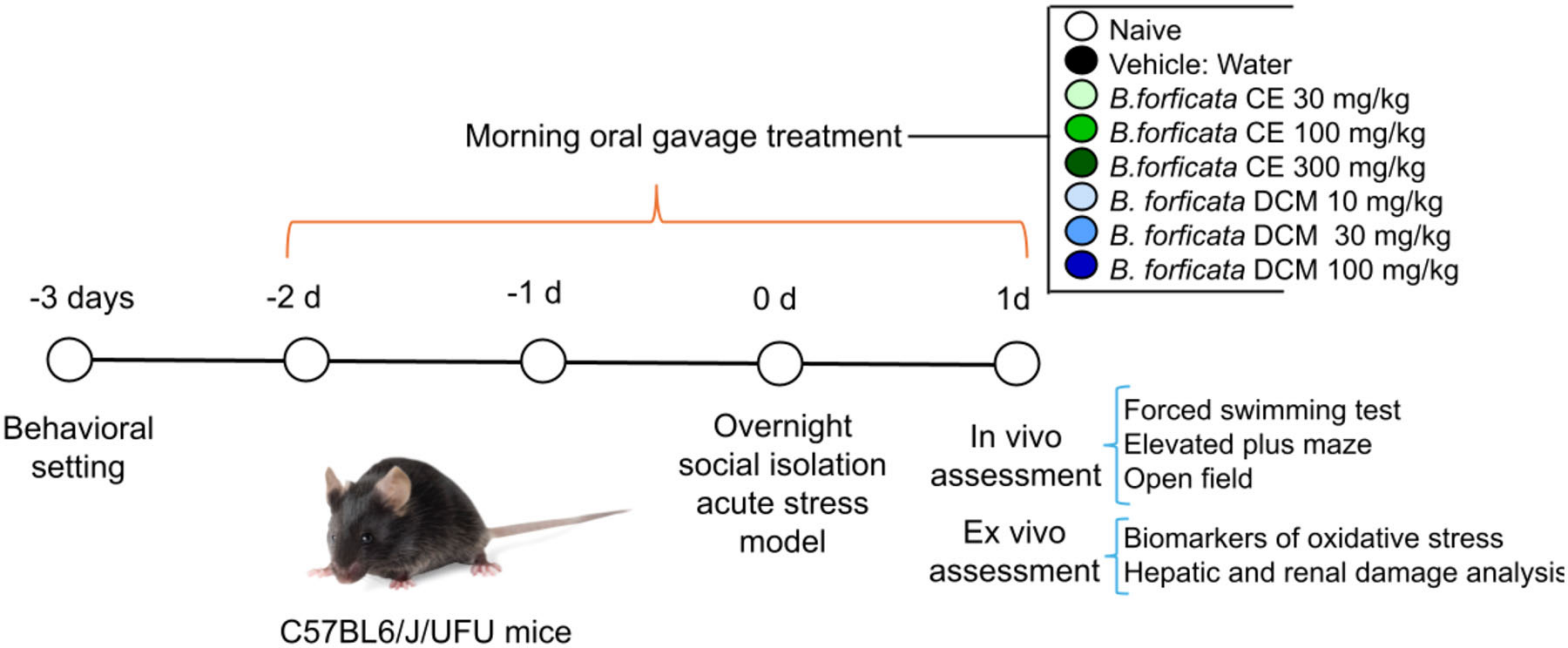
Experimental design.

Set 1: CE Evaluation (Franco et al. 2021; Tonelli et al. 2022)

*Naive*: Animals without stress or treatment.

*Vehicle*: Stressed animals treated with vehicle.

*CE 30 mg/kg*: Stressed animals treated with CE (30 mg/kg).

*CE 100 mg/kg*: Stressed animals treated with CE (100 mg/kg).

*CE 300 mg/kg*: Stressed animals treated with CE (300 mg/kg).

Set 2: DCM Fraction Evaluation (Franco et al. 2021).

*Naive*: Animals without stress or treatment.

*Vehicle*: Stressed animals treated with vehicle.

*DCM 10 mg/kg*: Stressed animals treated with DCM (10 mg/kg).

*DCM 30 mg/kg*: Stressed animals treated with DCM (30 mg/kg).

*DCM 100 mg/kg*: Stressed animals treated with DCM (100 mg/kg).

Both CE and DCM were freshly prepared daily by dilution in distilled water. Animals were treated by oral gavage once a day at 8:00 a.m., starting 2 days prior to stress induction and concluding on the day following stress exposure. This pretreatment schedule was designed to model a preventive approach, mimicking the preparation for impending stressful conditions.

Animals were assigned to groups by a pre‐established allocation scheme based on cage order. To prevent subjective bias, a double‐blind protocol was strictly maintained: the experimenters performing the daily oral administrations, conducting the behavioral assessments, and executing the ex vivo biochemical analyses were completely blinded to the treatment codes, which were only revealed during the final statistical analysis. Behavioral assessments were conducted manually by a single trained, blinded investigator to reduce inter‐observer variability. To further minimize potential variation, behavioral tests were performed on different days according to a predefined schedule. Prior to testing, mice were acclimatized to the room conditions and to the presence of the researcher.

### 2.4 Animal Behavioral Procedures

#### 2.4.1 Acute Stress Model

Animals were exposed to acute stress by social isolation animal model through overnight (12 h) social isolation in individual opaque cages (30 cm × 20 cm × 12.5 cm). During the overnight (12 h) period, they experienced water and food deprivation and were unable to make visual contact with others (Franco et al. 2021; Takatsu‐Coleman et al. 2013). The social isolation was done in a new experimental room, where the animals were not exposed before. Subsequently, the animals were regrouped in the same cages and returned to the experimental conditions they were used and subjected to behavioral analyses of the elevated plus maze, open‐field test, and forced swimming test.

#### 2.4.2 Forced Swimming Test

The mice were placed individually in the center of a glass cylindrical tank (17 cm × 25 cm) containing water (24°C–26°C) up to 10 cm (changed for each group) for a period of 6 min. The initial 2 min were intended to acclimatization, and in the remaining time, the duration of immobility (passive coping strategy) was recorded in seconds and considered indicative of depressive‐like behavior (Porsolt et al. 1977; Zanelati et al. 2010).

#### 2.4.3 Evaluation of the Development of Anxiety‐Like Behaviors

The elevated plus maze consisted of an apparatus with two open arms (without lateral walls) and two closed arms (with lateral walls). The mice were placed individually in the center of the platform with their heads facing the closed arm. They were allowed to freely explore the maze for 5 min. During this period, the duration of the animals’ stay in both the open and closed arms was timed and expressed in seconds (Campos et al. 2013).

#### 2.4.4 Assessment of Locomotor and Exploratory Activity

For the open‐field test, the animals were placed individually in the center of a circular arena (50 cm in diameter) containing symmetrical square markings on the floor and opaque walls, allowing the quantification of the animals’ locomotor activity. During 5 min, the number of crossings (times that the animal crossed each demarcated square with all four paws), the number of feces, and the time spent exploring the arena's periphery and center were observed (Franco et al. 2021).

### 2.5 Biochemical Analysis of Hepatic and Renal Damage

Blood samples were obtained via cardiac puncture 2 days after acute stress induction from three groups of mice: naive, CE (30 mg/kg), and DCM fraction (10 mg/kg). These doses were selected based on their protective effects against anxiety and depression, to reduce animals use, focusing on those that demonstrated therapeutic effects in most of the observed parameters. Serum was obtained by centrifugation at 3500 rpm for 10 min at 25°C, and hemoglobin concentration was determined. Levels below 180 mg/dL were considered suitable for subsequent biochemical analyses of alanine aminotransferase (ALT) and urea activity to evaluate the potential hepatic and renal damage from the treatments, respectively. All measurements were performed according to the Labtest Diagnostics commercial kit protocols and were analyzed using a Thermo Scientific Multiskan Go spectrophotometer.

### 2.6 Preparation of Nervous Tissue

After the behavioral tests, based on the protective effects against anxiety and depression, CE (30 mg/kg) and DCM (10 mg/kg) were selected for oxidative stress evaluation. The animals were euthanized by the administration of ketamine (300 mg/kg) and xylazine (50 mg/kg). Both hemispheres of the brain were divided into cortex, cerebellum, hippocampus, and striatum, which are central nervous system areas highly susceptible to oxidative damage and intrinsically related to anxiety and depression development. Subsequently, the samples were individually homogenized in pH 7.4 sodium phosphate buffer and centrifuged at 800 *g* and 4°C for 10 min. The supernatant was used to evaluate biomarkers of oxidative stress as previously described (Franco et al. 2021). Total protein concentration was assessed using the Bradford method with bovine serum albumin (BSA) as an analytical curve (Bradford 1976).

### 2.7 Oxidative Stress Biomarkers

#### 2.7.1 Superoxide Dismutase

Superoxide dismutase (SOD) activity was determined by inhibition of pyrogallol autoxidation, monitored kinetically at 420 nm. One unit is defined as 50% inhibition (Justino et al. 2017).

#### 2.7.2 Catalase

Catalase (CAT) activity was measured by H_2_O_2_ decomposition at 240 nm, monitored for 10 min in phosphate buffer (Justino et al. 2017).

#### 2.7.3 Glutathione Peroxidase

GR activity was assayed by monitoring NADPH consumption at 340 nm (Teixeira et al. 2017).

#### 2.7.4 Glutathione Reductase

The glutathione reductase (GR) activity was assessed by measuring NADPH consumption, using oxidized glutathione as a substrate. The homogenates were combined with 200 mM sodium phosphate buffer at pH 7.5, which included 6.3 mM EDTA to inhibit metal‐dependent enzymes, such as metalloproteases, 10 mM oxidized glutathione, and 3 mM NADPH. The decrease in the NADPH concentration was monitored for 10 min at 340 nm (Teixeira et al. 2017).

#### 2.7.5 Reduced Glutathione

GSH levels were quantified using a standard calibration curve. Reduced glutathione (GSH) was quantified fluorometrically (Ex 350/Em 420 nm) using ortho‐phthaldialdehyde after protein precipitation with metaphosphoric acid (Teixeira et al. 2017).

#### 2.7.6 Total Antioxidant Capacity (FRAP)

Ferric reducing ability was assessed by mixing samples with FRAP reagent (acetate buffer, TPTZ, FeCl_3_). Absorbance was read at 593 nm after 6 min at 37°C, using Trolox as a standard (Franco et al. 2018, 2021).

#### 2.7.7 Sulfhydryl Groups

Thiol groups (–SH) total sulfhydryl content was measured with DTNB in phosphate buffer (pH 8.0) at 412 nm (Aksenov and Markesbery^2001^.

#### 2.7.8 Lipid Peroxidation (TBARS)

Lipid peroxidation measuring malondialdehyde (MDA) levels were determined by the reaction with 0.67% thiobarbituric acid and measured fluorometrically (Ex 515/Em 553 nm) using MDA standard curves (Justino et al. 2017; Teixeira et al. 2017).

#### 2.7.9 Reactive Oxygen Species

To measure the ROS content, 20 µL of the homogenates were mixed with 10 µL of dichlorodihydrofluorescein diacetate (10 µM, diluted in ethanol) and 170 µL of 5 mM Tris‐HCl buffer (pH 7.4) and incubated for 3 min. After incubation was performed, the fluorescence intensity was measured in a 96‐well microplate at 530 nm (with excitation at 474 nm) (Justino et al. 2024).

### 2.8 Statistical Analysis

The sample size determination was performed using G*Power 3.1 software. An a priori power analysis indicated that a sample size of eight animals per group was required to achieve a statistical power of 80% (*α* = 0.05) for detecting significant effect sizes using one‐way analysis of variance (ANOVA). Statistical analyses and graphic visualization were performed in GraphPad Prism 8.0 software. Prior to parametric analyses, data normality was verified using the Shapiro–Wilk test, and homogeneity of variance was assessed using the Brown–Forsythe test. Because the assumptions for parametric testing were met, the differences between groups were analyzed using one‐way ANOVA followed by Dunnett's post hoc test. Data are expressed as mean ± standard error of the mean (SEM). *p* values < 0.05 were considered statistically significant.

## 3 Results

### 3.1 Liquid Chromatography‐Mass Spectrometry Analysis

HPLC‐ESI‐MS/MS analysis allowed the tentative assignment of ten compounds in the DCM fraction (Table 1), including the flavonoid rutin (*m/z* 609 [M–H]^−^) as well as other quercetin‐ and kaempferol‐derived glycosides, phenolic acids, and lupeol. These compounds, in special rutin and kaempferol‐derived glycosides, have been previously related with anxiolytic and antidepressive activities in different animal models, as well as antioxidant potential (Anesti et al. 2020; Foudah et al. 2022; Ren et al. 2019; dos Santos et al. 2021). Consequently, their prominent presence in the DCM fraction provides a putative phytochemical basis for the behavioral resilience and the specific regional redox modulation observed in the subsequent in vivo and ex vivo assays of this study. These metabolites were putatively annotated based on accurate mass measurements and MS/MS fragmentation patterns, through comparison with literature data, without confirmation using authentic standards. Figure 2 presents the proposed chemical structures of these compounds, while Table 1 summarizes the corresponding LC‐MS data.

**FIGURE 2:**
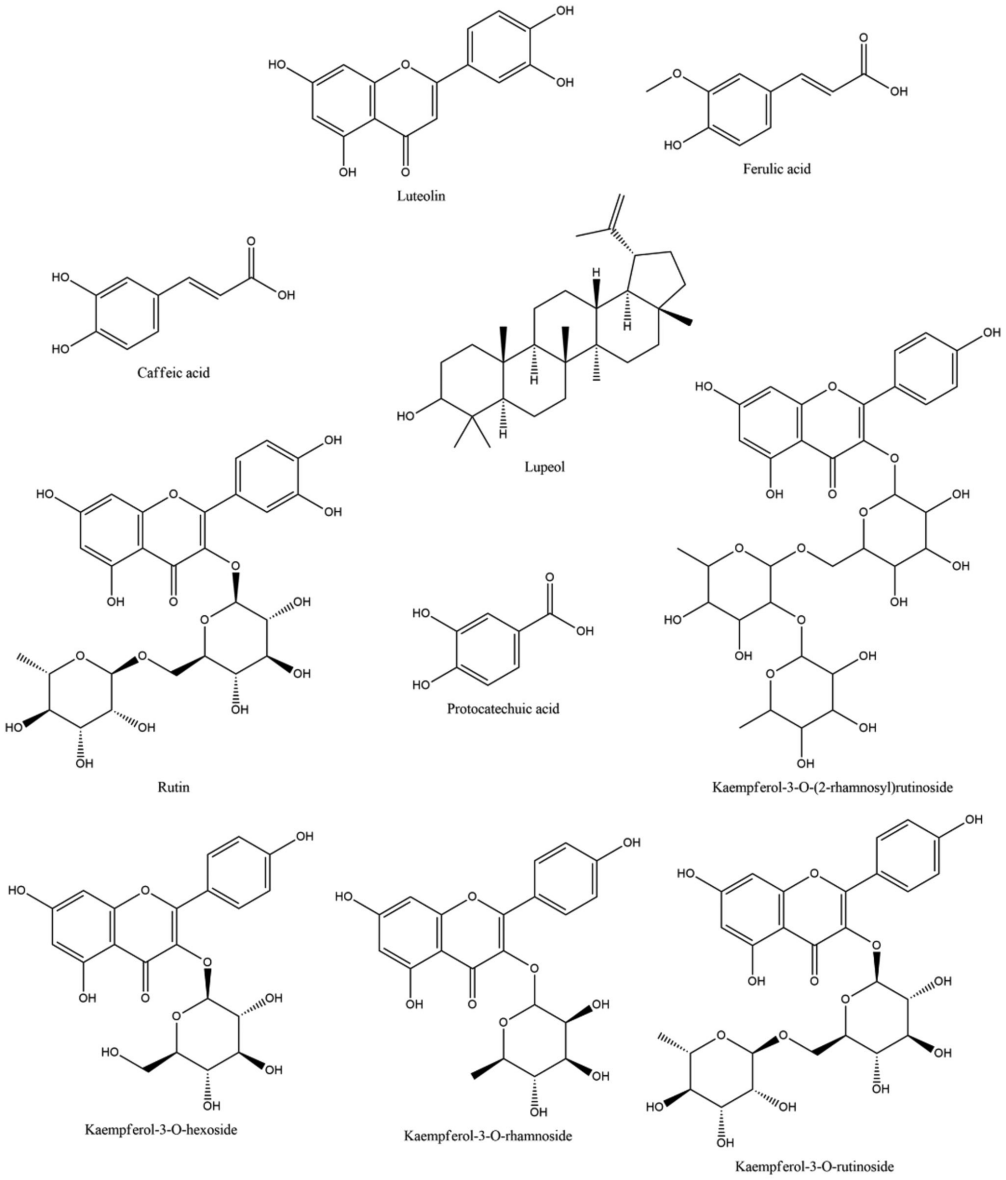
The molecular structure of metabolites putatively present in the dichloromethane fraction of leaves. Compound identification was based on retention time, exact mass, comparison of calculated and observed *m/z* values, MS/MS fragmentation profiles, and reference to spectral libraries and published studies. A mass error threshold of ≤ 10 ppm was applied.

### 3.2 Protective Effect of the Crude Extract and Dichloromethane Fraction of *B. forficata* Leaves on Depressive‐Like Behaviors

The animals subjected to acute stress induced by social isolation and treated only with the vehicle exhibited a significant increase of 74.6 s (105 ± 7% increase) in immobility during the forced swim test compared to naive animals, which is indicative of depressive‐like behaviors. Conversely, stressed animals treated with CE of *B. forficata* (30, 100, and 300 mg/kg) demonstrated a reduction in immobility time of 58.1 s (40 ± 8%), 53.2 s (36 ± 8%), and 30.9 s (22 ± 3%), respectively, compared to the vehicle‐treated group (Figure 3A). Notably, the highest dose (300 mg/kg) exhibited a noticeably weaker effect compared to the lower doses, indicating an inverted U‐shaped dose‐response pattern. This finding indicates that *B. forficata* CE treatment prevented the development of depressive‐like behavior in the stressed mice.

**FIGURE 3:**
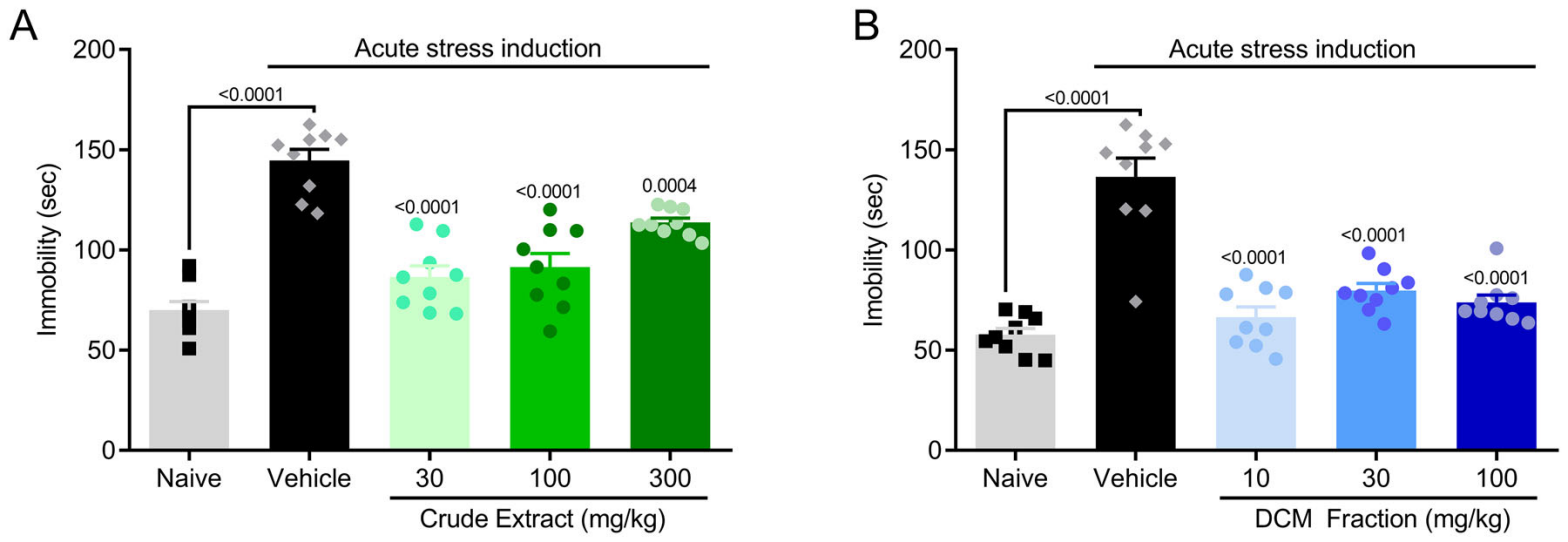
Crude extract and dichloromethane fraction of *B. forficata* mitigate depressive‐like behavior in animals subjected to social isolation‐induced stress. Immobility time of naive animals and animals subjected to social isolation and treated with vehicle, the crude extract of *B. forficata* at doses of 30, 100, and 300 mg/kg, or the dichloromethane fraction at doses of 10, 30, and 100 mg/kg. Assessments were conducted over 4 min in a water‐filled cylinder at 26°C. *N* = 9 animals/group. Dunnett‐adjusted *p*‐values for significant comparisons of both the naive and the treated groups relative to the vehicle group are displayed directly above the respective bars. Data are expressed as mean ± SEM with individual data points. Statistical analysis was performed using one‐way ANOVA followed [Panel (A): *F*(4, 40) = 32.68, *p* < 0.0001; Panel (B): *F*(4, 40) = 32.97, *p* < 0.0001] by Dunnett's post‐test.

Furthermore, the DCM fraction of *B. forficata* also exhibited a preventive effect against depressive‐like behavior development in animals during the forced swim test. Stressed animals treated with DCM at 10, 30, and 100 mg/kg displayed a reduced immobility time of 70.0 s (51 ± 9%), 56.8 s (45 ± 6%), and 62.7 s (41 ± 7%), respectively, compared to the stressed group treated with vehicle (Figure 3B).

### 3.3 Protective Effect of Crude Extract and Dichloromethane Fraction of *B. forficata* on Anxiety‐Like Behavior

Animals subjected to the social isolation‐induced acute stress model and treated with vehicle exhibited a reduced duration of 51.6 s (39 ± 6% reduction) of permanence in the open arms, accompanied by an increased duration of 45.5s (26 ± 7% increase) spent in the closed arms in comparison to naive animals, which was indicative of anxiety‐like behaviors. Conversely, only animals treated with the lower dose of CE, 30 mg/kg, were protected from this reduction of 40.9 s (80 ± 8% of protection) spent in the open arms, and, consequently, were also protected from spending 44.8 s (93 ± 6% of protection) in the closed arms, when compared to stressed animals treated with vehicle (Figures 4A,B). Notably, the higher doses (100 and 300 mg/kg) did not significantly differ from the stressed control in elevated plus maze measures. In contrast, pretreatment with the DCM fraction revealed a distinct and broader anxiolytic‐like profile; all evaluated doses (10, 30, and 100 mg/kg) were able to protect against the decreased time spent in the open arms by 51.5 s (98 ± 12%), 40.6 s (77 ± 9%), and 40.2 s (77 ± 13%), respectively, and increased time in closed arms, when compared to the vehicle‐treated stressed animals.

**FIGURE 4:**
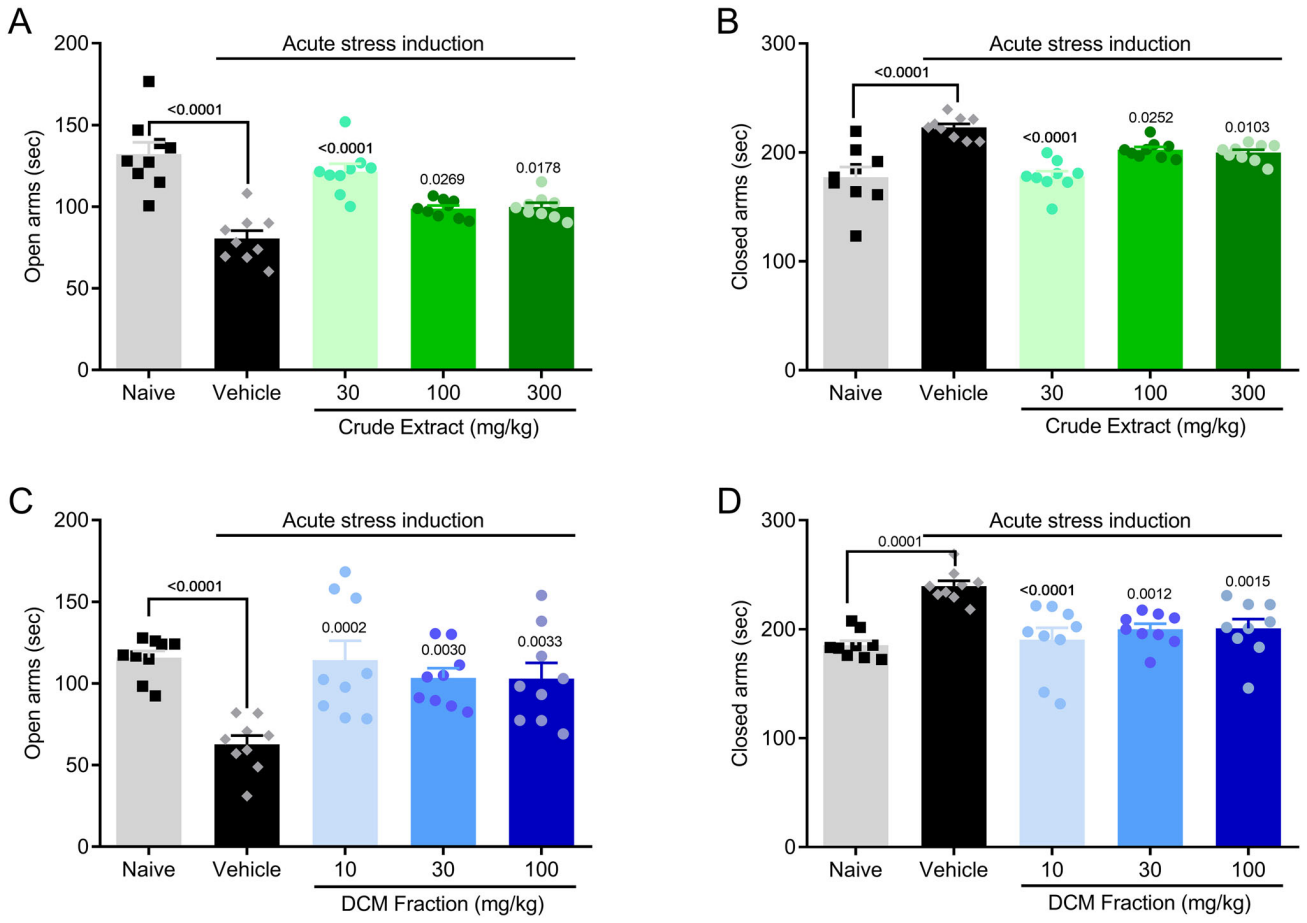
Crude extract (A, B) and dichloromethane fraction (C, D) of *B. forficata* reduce anxiety‐like behavior in animals subjected to social isolation‐induced acute stress. Time spent in the open arms (A, C) and closed arms (B, D) of the elevated plus maze for naive animals and mice subjected to social isolation and treated with the vehicle, crude extract of *B. forficata* at doses of 30, 100, and 300 mg/kg, or the dichloromethane fraction at doses of 10, 30, and 100 mg/kg. *N* = 9 animals/group. Dunnett‐adjusted *p*‐values for significant differences between the vehicle group and naive animals, as well as between the treated groups and the vehicle group, are displayed directly above the respective bars. Data are expressed as mean ± SEM with individual data points. Statistical analysis was performed using one‐way ANOVA [Panel (A): *F*(4, 40) = 19.38, *p* < 0.0001; Panel (B): *F*(4, 40) = 13.72, *p* < 0.0001; Panel (C): *F*(4, 40) = 7.411, *p* = 0.0001; Panel (D): *F*(4, 40) = 8.972, *p* < 0.0001] followed by Dunnett's post‐test.

Open field test analysis resulted in the observation of a clear reduction in 36.6 s (62 ± 8% reduction) spent in the central region and a corresponding increase of the same time (15 ± 8% increase) in the time spent in the peripheral region by untreated animals subjected to the stress model when compared to naive animals (Figures 5A,B). Additionally, there was a higher number of 3.5 fecal pellets (158 ± 16% of increase) in the stressed group treated with vehicle than in the naive group, which is also indicative of anxiety (Figure 5C).

**FIGURE 5:**
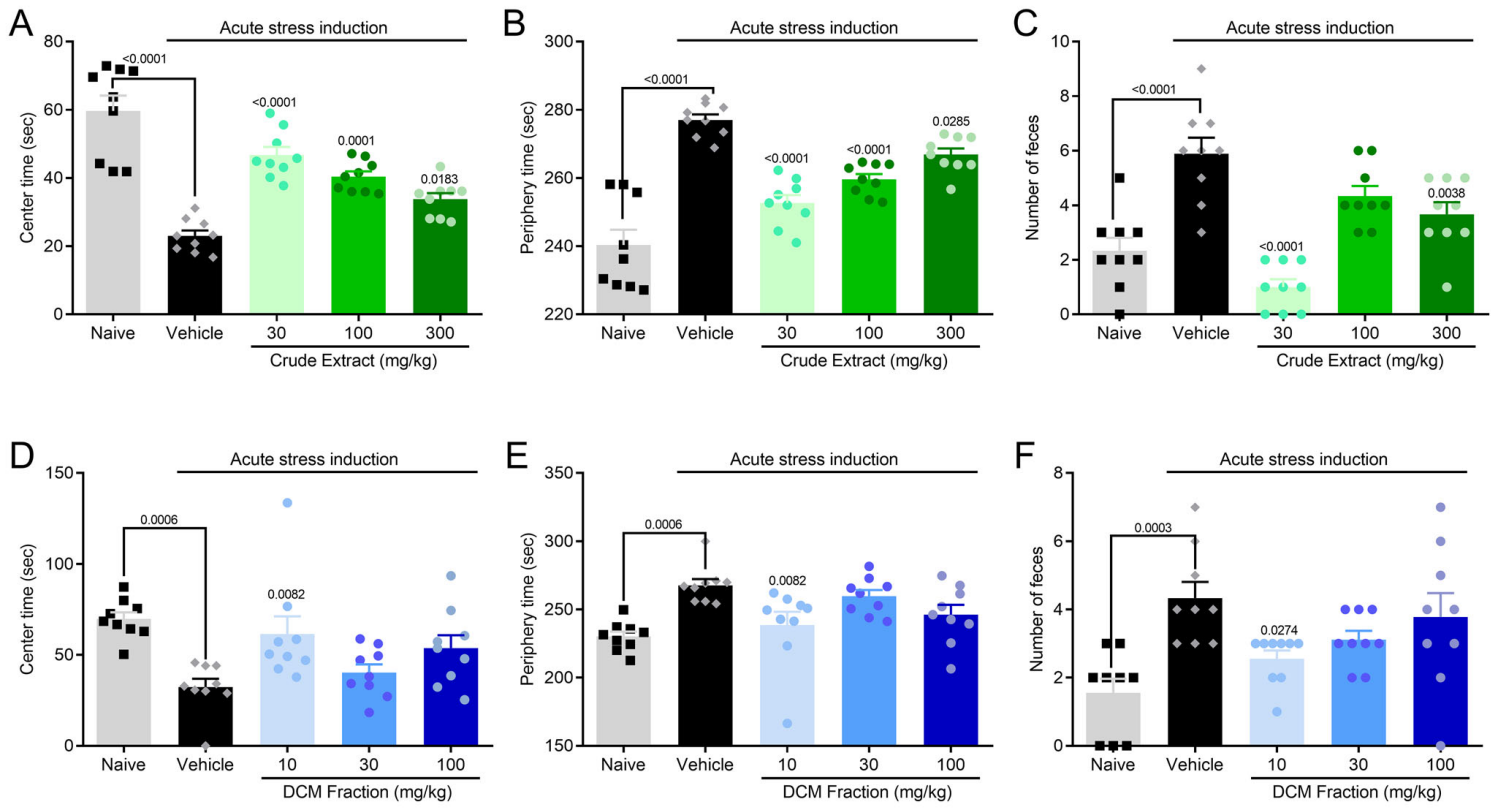
Crude extract (A–C) and dichloromethane fraction (D–F) of *B. forficata* mitigate anxiety‐like behavior induced by social isolation‐induced acute stress model. Open Field Method was conducted over 5 min, measuring three distinct parameters: Time spent in the center (A, D), time spent in the periphery (B, E), and number of fecal pellets (C, F) for naive animals, and stressed animals treated with vehicle, 30, 100, and 300 mg/kg of crude extract or 10, 30, and 100 mg/kg of a dichloromethane fraction obtained from the leaves of *B. forficata*. *N* = 9 animals per group. Dunnett‐adjusted *p*‐values for significant differences between the vehicle group and naive animals, as well as between the treated groups and the vehicle group, are displayed directly above the respective bars. Data are expressed as mean ± SEM with individual data points. Statistical analysis was conducted using one‐way ANOVA [Panel (A): *F*(4, 40) = 28.32, *p* < 0.0001; Panel (B): *F*(4, 40) = 29.06, *p* < 0.0001; Panel (C): *F*(4, 40) = 17.81, *p* < 0.0001; Panel (D): *F*(4, 40) = 5.842, *p* = 0.0008; Panel (E): *F*(4, 40) = 5.842, *p* = 0.0008; Panel (F): *F*(4, 40) = 5.750, *p* = 0.0009] followed by Dunnett's post‐test.

When comparing stressed vehicle‐treated groups against stressed CE treatments across all parameters, the lower dose of 30 mg/kg significantly protected against stress‐induced alterations. Specifically, for time spent in the center, the 30, 100, and 300 mg/kg doses increased exploration by 23.8 s (70 ± 7%), 17.4 s (47.4%), and 10.8 s (29.4%), respectively, compared to the vehicle group. For time spent in the periphery, the treatments attenuated thigmotaxis by 24.3 s (67 ± 3%), 17.4 s (47.4%), and 10.1 s (27.5%), respectively. Finally, regarding the number of fecal pellets, the CE doses reduced defecation by 4.9 pellets (135 ± 11%), 1.6 pellets (45.0%), and 2.2 pellets (61.9%), respectively, when compared to the vehicle‐treated stressed animals.

Collectively, the data demonstrate a bell‐shaped efficacy profile for the CE. The anxiolytic‐like protection achieved at 30 mg/kg was partially lost at 100 mg/kg and completely abolished at the highest dose of 300 mg/kg, indicating a restricted optimal dosing window (Figures 5A–C). Furthermore, the dose of CE 100 mg/kg also protected from the reduction on the time spent in the center, and the increased time spent in the periphery, but not from increased numbers of feces compared to the vehicle group (Figures 5A–C).

DCM‐treated animals were protected across all observed parameters; however, only with the lower dose of 10 mg/kg. This dose led to an increase in the time spent in the center by 29.2 s (78 ± 16%), a reduction in the time spent in the periphery by 29.2 s (79 ± 6%), and a decrease in the number of feces by 1.8 pellets (64 ± 5%) when compared to the vehicle group (Figure 5D–F). Doses of 30 and 100 mg/kg of DCM did not demonstrate protective effects on these parameters. These findings suggest that oral administration of DCM, in the dose of 10 mg/kg, mitigated the development of anxiety‐like behaviors in stressed mice.

Regarding crossing behavior, stress resulted in an increase of 45 number of crossings activities (56 ± 9% increase) compared to the naive group, reflecting stress‐induced hyperarousal and hyperlocomotion typical of escape‐like behaviors in acute stress models. CE treatment with doses of 30 and 100 mg/kg significantly protected against the stress‐induced increase in crossing activity, reducing the number of crossings by 33.1 (73.5%) and 30.2 (67.1%), respectively. Interestingly, the highest CE dose of 300 mg/kg did not significantly protect against this hyperlocomotion, consistent with the bell‐shaped efficacy observed in other parameters. Furthermore, in the DCM experimental set, treatment with doses of 10, 30, and 100 mg/kg reduced the crossing activities by 31.2 (101.0%), 18.0 (58.3%), and 20.4 (66.0%), respectively. Notably, the doses of 10 and 100 mg/kg effectively protected against the increased crossing activities compared to the vehicle group (Figure 6A,B). Interestingly, the higher dose of CE, 300 mg/kg, as in other parameters, did not protect from the increase in crossing activities.

**FIGURE 6:**
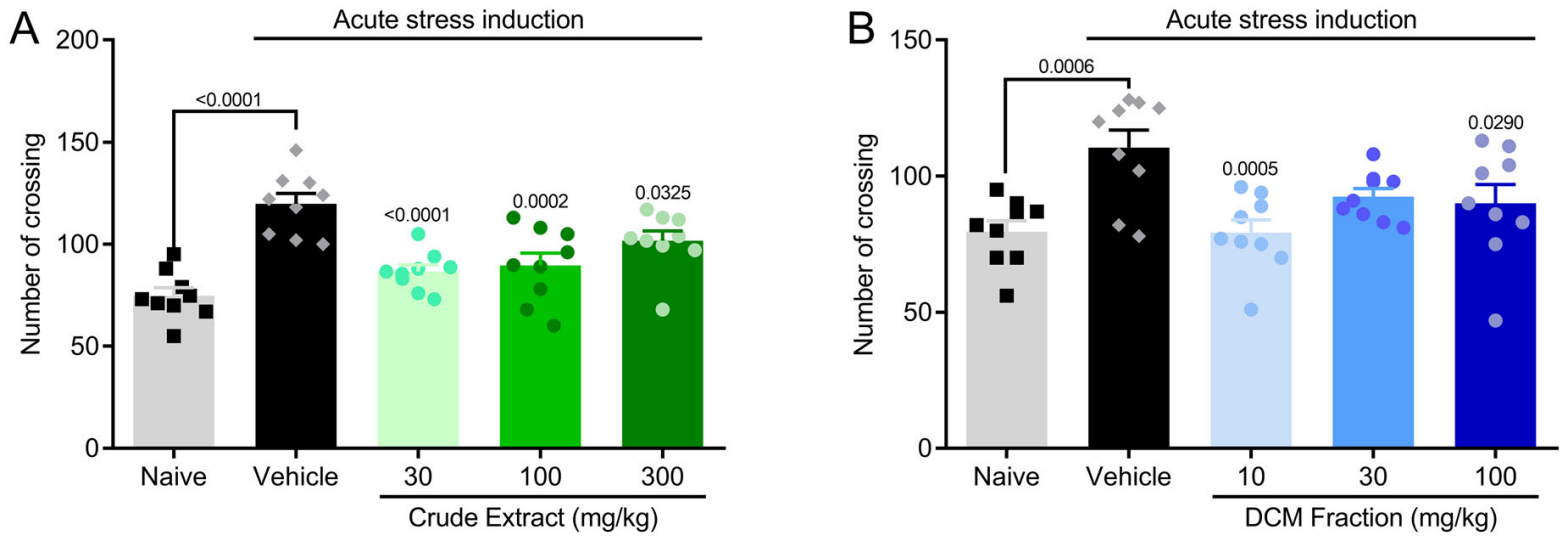
Effect of the crude extract (A) and dichloromethane fraction (B) of *B. forficata* on crossing behavior in animals subjected to social isolation‐induced acute stress model. Number of crossings in the open field test observed in naive animals, stressed group treated with vehicle, and stressed groups treated with vehicle, 30, 100, and 300 mg/kg of crude extract or 10, 30, and 100 mg/kg of the dichloromethane fraction obtained from the leaves of *B. forficata*. *N* = 9 animals per group. Dunnett‐adjusted *p*‐values for significant differences between the vehicle group and naive animals, as well as between the treated groups and the vehicle group, are displayed directly above the respective bars. Data are expressed as mean ± SEM with individual data points. Statistical analysis was conducted using one‐way ANOVA [Panel (A): *F*(4, 40) = 13.19, *p* < 0.0001; Panel (B): *F*(4, 40) = 5.959, *p* = 0.0007] followed by Dunnett's post‐test.

**FIGURE 7:**
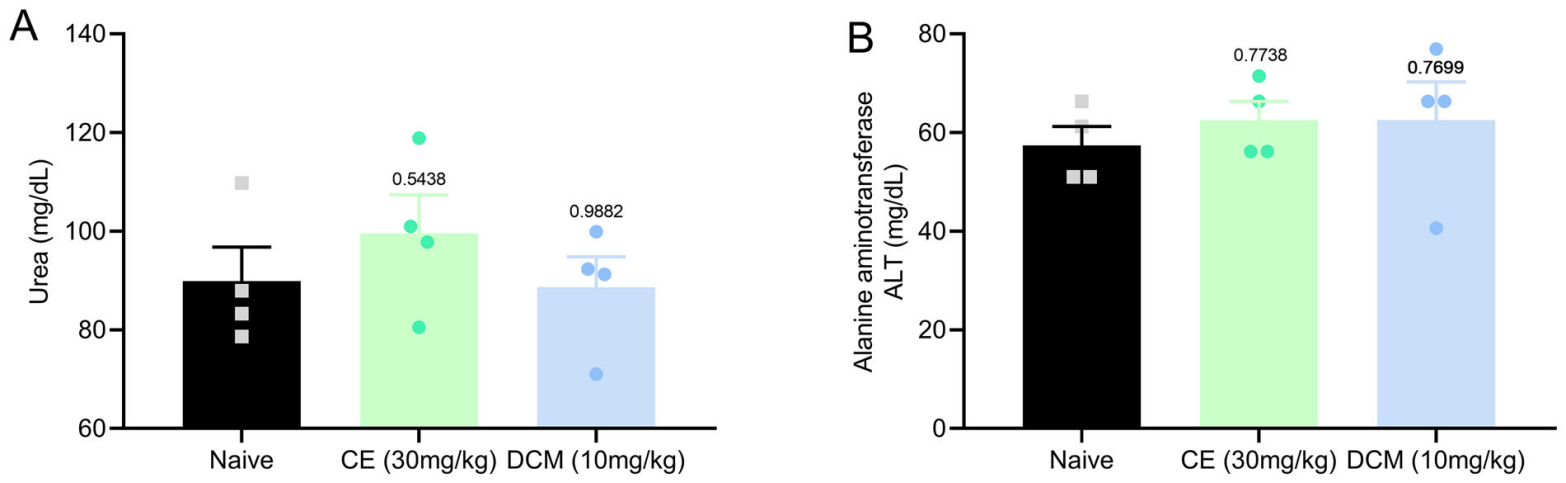
Crude Extract and dichloromethane fraction of *Bauhinia forficata* show no impact on urea levels and ALT activity. Comparison of serum urea concentration (A) and alanine aminotransferase (ALT) enzyme activity (B) between naive animals and mice treated with the crude extract at the dose of 30 mg/kg for the CE, and 10 mg/kg for DCM fraction. *N* = 4 animals/group. Dunnett‐adjusted *p*‐values for significant differences between the vehicle group and naive animals, as well as between the treated groups and the vehicle group, are displayed directly above the respective bars. Data are expressed as mean ± SEM with individual data points. Statistical analysis was conducted using one‐way ANOVA [Panel (A): *F*(2, 9) = 0.7239, *p* = 0.5111; Panel (B): *F*(2, 9) = 0.2954, *p* = 0.7512] followed by Dunnett's post‐test.

Therefore, 30 mg/kg CE and 10 mg/kg DCM fraction, representing the minimum effective doses, were used for the subsequent ex vivo analyses since they produced the most pronounced and comprehensive protective effect across all evaluated behavioral endpoints, such as rescuing open‐arm exploration in the elevated plus maze, normalizing central locomotion and thigmotaxis in the open field test, and reducing immobility time in the forced swim test. Interestingly, higher doses did not demonstrate the same broad protective effects in the evaluated parameters.

### 3.4 Hepatic and Renal Function After Oral Administration of *B. forficata*

There were no significant differences seen in the serum levels of urea and ALT between the naive animals and mice treated with 30 mg/kg of the CE or 10 mg/kg of the DCM fraction of *B. forficata*, indicating that these specific therapeutically effective doses did not induce overt hepatic or renal damage (Figure 7).

### 3.5 Antioxidant Effect of the Crude Extract and Dichloromethane Fraction of *B. forficata* on Different Brain Regions

The effects of the treatments on oxidative stress biomarkers across the different brain regions are visually summarized in the heatmap (Figure 8), with all absolute quantitative data and precise statistical comparisons detailed in Table S1. As this overview reveals, acute stress did not produce a uniform oxidative response; rather, it induced a highly region‐specific redox profile. Specifically, the striatum emerged as the primary target of oxidative disruption, whereas other regions displayed distinct baseline alterations and adaptive adjustments, providing a clear anatomical context for the specific modulatory effects of CE and DCM.

**FIGURE 8:**
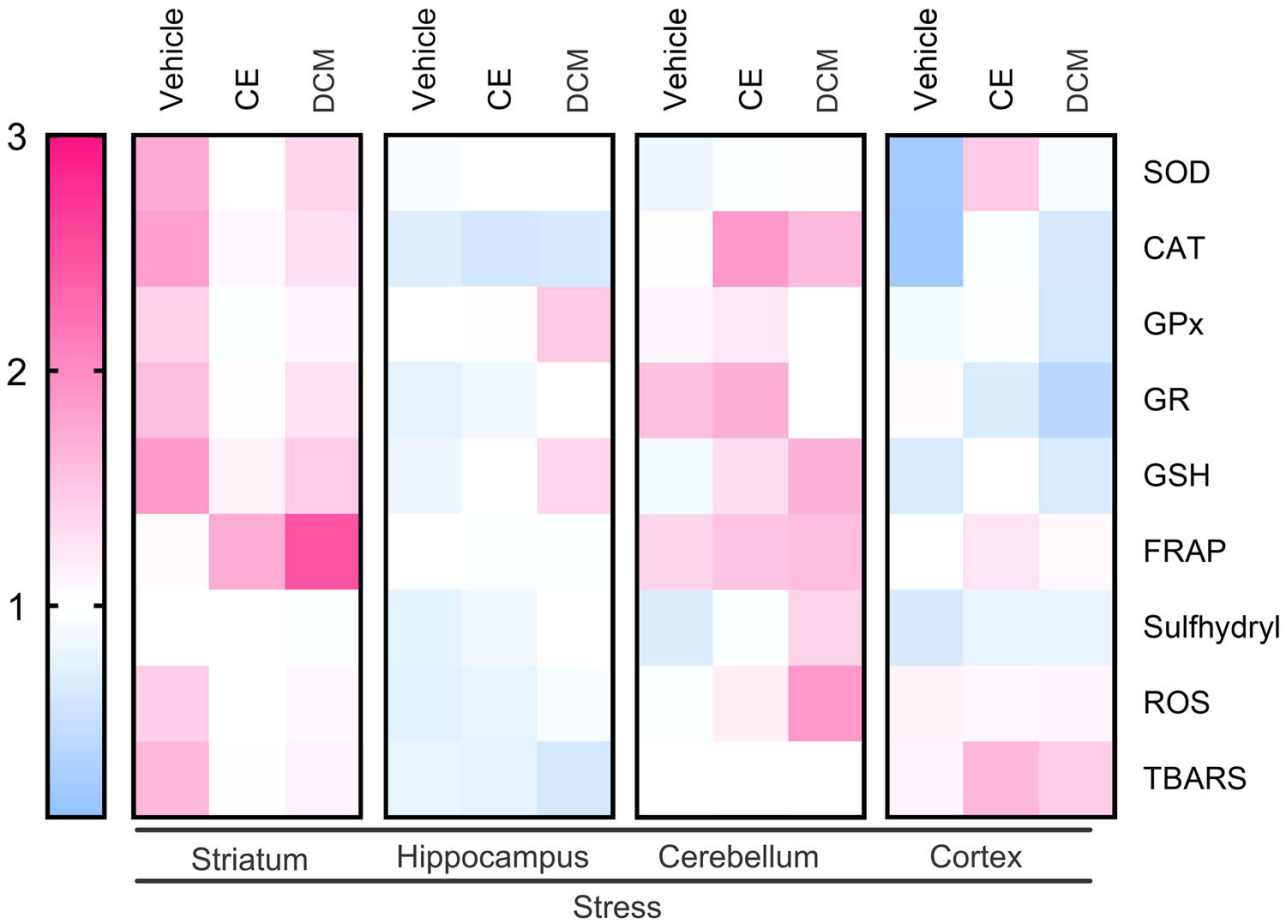
Heatmap of the changes in oxidative stress biomarkers in brain regions of animals treated with *Bauhinia forficata* crude extract and dichloromethane fraction. The heatmap illustrates the relative changes in the levels of oxidative stress biomarkers. Data are expressed as a ratio relative to the naive (control) group, which was normalized to a value of 1 (represented by the color white). The intensity of the pink/red color indicates a proportional increase in the biomarker levels compared to the naive group, while the intensity of the blue color indicates a proportional decrease. The rows represent the analyzed biomarkers: superoxide dismutase (SOD), catalase (CAT), glutathione peroxidase (GPx), glutathione reductase (GR), glutathione (GSH), ferric reducing antioxidant power (FRAP), sulfhydryl, reactive oxygen species (ROS), and lipid peroxidation (TBARS). The columns represent the four brain regions (striatum, hippocampus, cerebellum, and cortex) and the respective treatment groups: stressed animals treated with vehicle (Vehicle), with 30 mg/kg of CE, or with 10 mg/kg of the DCM fraction. *N* = 8 animals/group. Statistical analysis was conducted using one‐way ANOVA followed by Dunnett's post‐test. Detailed quantitative data, including absolute values (mean ± SEM) and exact statistical *p*‐values for all biomarkers, are provided in Table S1.

In the striatum, the vehicle‐treated stress group exhibited a significant increase in oxidative damage markers compared to the naive group, specifically elevating ROS from 986.1 ± 94.8 to 1414.3 ± 169.4 units of fluorescence (a 43.4% increase) and TBARS from 1.05 ± 0.085 to 1.71 ± 0.30 nmol MDA/mg (a 62.9% increase). This was accompanied by a compensatory upregulation of antioxidant defenses (SOD, CAT, glutathione peroxidase [GPx], GR, and GSH). Treatment with the CE prevented this oxidative challenge by reducing ROS by 450.5 units of fluorescence (reaching 963.7 ± 32.1; a 31.9% reduction compared to vehicle) and TBARS by 0.62 nmol MDA/mg (reaching 1.09 ± 0.04; a 36.3% reduction), thereby preventing the need for the compensatory increase in antioxidant enzymes and effectively normalizing their levels toward the naive baseline. Notably, the CE treatment also caused an increase in total antioxidant capacity (FRAP) to 70.9 ± 12.3 µmol trolox eq/mg (a 61.2% increase) compared to the vehicle group (44.0 ± 5.4 µmol trolox eq/mg). Similarly, the DCM fraction treatment markedly increased FRAP levels to 101.4 ± 3.1 µmol trolox eq/mg (a 130.6% increase) while also reducing TBARS levels by 0.54 nmol MDA/mg (reaching 1.17 ± 0.10; a 31.6% reduction) relative to the vehicle group.

In the hippocampus, vehicle‐treated animals showed a reduction in the levels of CAT, GR, sulfhydryl, ROS, and TBARS compared to the naive group. The CE treatment did not produce significant changes for these markers when compared to the vehicle group. In contrast, the DCM fraction treatment increased the levels of the antioxidants GPx, GR, GSH, and sulfhydryl relative to the vehicle group.

In the cerebellum, no significant differences were observed between the naive and vehicle‐treated groups. However, DCM treatment led to a significant increase in ROS, GSH, and sulfhydryl in the cerebellum relative to stressed controls. Furthermore, treatment with CE led to an increase in CAT activity.

Finally, in the cortex, the vehicle group exhibited reduced SOD and CAT activity, along with lower sulfhydryl concentrations, compared to the naive group. The CE treatment effectively prevented this decline, maintaining higher SOD and CAT activity relative to the vehicle group. Conversely, the DCM fraction treatment led to a reduction in GR activity compared to the vehicle group.

## 4 Discussion

Stress manifestation includes a diverse array of physical, emotional, and psychological symptoms, and prolonged exposure to stressors without appropriate intervention can culminate in severe health problems, such as anxiety and depression (Chu et al. 2025). These conditions impose a significant socioeconomic burden and profoundly disrupt individuals' lives. For these reasons, options to relieve adverse stress symptoms and allow an easier adaptation to stress are still being investigated, including the use of natural products with adaptogenic potential (Franco et al. 2021; Panossian 2017; Speers et al. 2021). Thus, given *B. forficata*’s well‐documented antioxidant, hepatoprotective, cardioprotective, anti‐hyperglycemic, and anti‐inflammatory activities, commonly described in adaptogens, we investigated whether its leaf extracts could alleviate stress‐induced anxiety and depressive‐like behaviors in mice (Gudavalli et al. 2024b; Panossian and Wikman 2010). Sustaining these effects, we also highlighted a protective role of this plant against oxidative stress in specific brain regions associated with anxiety and depression.

The adaptogenic effects of plants are often attributed to their secondary metabolites (terpenoids, phenolics, etc.), leading to antioxidant and anti‐inflammatory actions (Sánchez et al. 2023; Shitan 2016). Consistent with this, our phytochemical analysis suggests that *B. forficata* is rich in flavonoids including rutin and quercetin/kaempferol derivatives. These compounds were tentatively identified based on LC–MS/MS data. Because they are widely recognized in the literature for their adaptogenic‐like, antioxidant, and neuroprotective properties (Anesti et al. 2020; Emudainohwo et al. 2023; Foudah et al. 2022; Lee et al. 2020; Oyovwi et al. 2025; Ren et al. 2019; dos Santos et al. 2021; Silva dos Santos et al. 2021), their tentative presence in *B. forficata* leaf extracts may suggest potential mechanistic pathways underlying the anti‐stress effects observed in this study. However, further studies evaluating these isolated compounds should be conducted to confirm their individual effects.

Interestingly, the adaptogenic‐like effects of *B. forficata* were most pronounced at lower doses (CE 30 mg/kg and DCM 10 mg/kg), with higher doses not increasing efficacy and, in some tests, showing lesser effect. The reduced efficacy at higher doses likely may result from pharmacokinetic limitations, or, considering the different compounds found in plants extracts, low doses might positively influence beneficial pathways, while high doses can activate harmful ones. Additionally, there are no changes in ALT or urea, suggesting an absence of liver or kidney damage at the tested doses. This safety profile is key for adaptogens, which should not harm normal physiological functions.

Corroborating this adaptogenic‐like potential, both CE and DCM fractions effectively protected against depressive‐like behaviors, reducing immobility time in the forced swim test, a common indicator of despair (Xu et al. 2025; Yousuf et al. 2020). Although our analysis tentatively identified rutin and derivatives of kaempferol and quercetin in the extracts, it is notable that kaempferol and quercetin themselves are reported to reduce immobility in both the tail suspension and forced swim tests in previous studies (Park et al. 2010). Rutin alone has antidepressive action described in rats exposed to an unpredictable chronic stress paradigm, also in a reserpine‐induced depression animal model (Chew et al. 2012; Foudah et al. 2022). Therefore, reserpine‐induced depression by monoamine depletion in animals has been proven to be a correlation for studying depression in humans (Baumeister et al. 2003).

Regarding the anxiolytic effect, both treatment groups effectively reduced anxiety‐like symptoms observed in the plus‐maze apparatus by reducing time spent in the closed arms, a behavioral trait observed when animals experience stress and anxiety‐like behavior, leading them to avoid open, unfamiliar, and potentially hazardous environments, which is consistent with the findings of previous studies (Choleris 2001; Franco et al. 2021; Walf and Frye 2007). Literature previously pointed out rutin as a prominent anxiolytic compound, able to normalize the hypothalamic–pituitary–adrenal axis, which is the key regulator of stress, besides having important antioxidant properties (Emudainohwo et al. 2023). Interestingly, animal models of social isolation stress, particularly in early life, are known to activate the hypothalamic–pituitary–adrenal axis and interact with monoaminergic, glutamatergic, and GABAergic neurotransmitter systems, inducing long‐lasting reductions in serotonin turnover and alterations in dopamine receptor sensitivity, and have been implicated in addictive, psychotic, and affective disorders (Brandt et al. 2022).

Additionally, molecular docking studies demonstrated that rutin shows promising interactions with the GABAB receptor (Karpuz Ağören et al. 2024). Quercetin also protects against anxiety‐like symptoms, and these effects were attributed to the anti‐inflammatory properties on hippocampus, reducing the release of cytokines like IL‐1B and IL‐6 (Lee et al. 2020; Oyovwi et al. 2025). Flavonoids can counteract stress‐induced imbalances on neurotransmitters such as dopamine, norepinephrine, serotonin, and gamma‐aminobutyric acid (GABA) critical on the onset of mood disorders (Bouras et al. 2023; Foudah et al. 2022; Khan et al. 2018). While our study did not directly measure HPA axis hormones or inflammatory cytokines, the known actions of the identified flavonoids suggest these pathways could be involved in *B. forficata's* effects. Measuring cortisol (or corticosterone in rodents) levels, or cytokine profiles, in future experiments would be valuable to confirm these mechanistic links.

Furthermore, in the open field test, after treatment, the animals showed a reduction in the number of crossings and feces as well as a decrease in the time spent in the periphery, suggesting less anxious and stressful behavior, once animals displaying anxious and stress‐like behaviors often defecate more frequently and spend prolonged periods in the peripheral regions of the field (Çevik et al. 2025; Choleris 2001). Although the open field test is primarily indicative of locomotor activity, it has also been widely employed to evaluate anxiety‐ and depression‐like behaviors. This test is quite sensitive for assessing anxiety based on the conflict between exploration and aversion to open environments, with less time spent in the center/more time spent in the periphery being described as thigmotaxis, suggesting greater anxiety. Importantly, the extracts normalized crossing behaviors without suppressing them below naive levels or acting as general motor stimulants, which may suggest that the observed anxiolytic‐ and antidepressant‐like effects are genuine and not confounded by sedation or hyperactivity, respectively. Thus, for the first time demonstrated, treatment with *B. forficata* has shown protective effects against stress‐induced anxiety and depression, supporting its potential role as a new adaptogenic‐like medicinal herb.

Inflammation and oxidative stress have been implicated in the pathogenesis of some neuropsychiatric disorders, such as anxiety and depression, with the brain being uniquely susceptible to oxidative stress, demonstrating decreased antioxidant defenses combined with increased oxidative damage in these situations (Fedoce et al. 2018; Özdemir and Demir 2026; Speers et al. 2021). Therefore, we focused our analysis on brain areas involved with anxiety, depression, and stress behaviors, also related to oxidative stress (Adhikari et al. 2010; Floyd and Hensley 2002; Pandya et al. 2012).

Our findings support a region‐dependent redox framework for the effects of acute stress and *B. forficata* treatment. Rather than producing a uniform oxidative response across the brain, acute stress appears to differentially engage hippocampal, cortical, cerebellar, and striatal pathways according to their intrinsic metabolic activity, mitochondrial function, basal ROS production, and antioxidant capacity (Anusha‐Kiran et al. 2022; Vinokurov et al. 2021). Within this framework, the striatum emerged as the main target of oxidative disruption and also the principal site of protection by the CE and DCM fraction, which is neurobiologically relevant because oxidative imbalance in this region can interfere with neurotransmitter systems involved in motivation, emotional regulation, and stress‐related behavioral output (Hovatta et al. 2010; Wang et al. 2025). In parallel, the increase in cortical SOD and catalase and the enhancement of hippocampal GR and sulfhydryl groups suggest recruitment of endogenous antioxidant defenses, while the reduction in hippocampal ROS and TBARS is consistent with an early adaptive response that restores redox balance rather than reflecting absence of stress effects (Salim 2017). The slight increase in cerebellar ROS after DCM treatment, although initially unexpected, may reflect persistent oxidative signaling in a region with high basal ROS activity and distinct redox physiology, rather than overt injury (Venkateshappa et al. 2012; Vinokurov et al. 2021).

The striatum controls cognition, learning, and motivated behavior and was the most affected by oxidative stress after social isolation (Macpherson and Hikida 2019). On striatum, we also identified the most prominent *B. forficata* protective effects against oxidative stress damage, where the CE treatment lowered the increase of different oxidative indicators, and both CE and DCM fraction prevented the compensatory upregulation of antioxidant enzymes by reducing the underlying oxidative challenge, as evidenced by the reduction in ROS and lipid peroxidation (TBARS). Literature indicates that quercetin and kaempferol derivatives, such as those tentatively annotated in our extracts, possess recognized antioxidant properties, such as reduction of TBARS levels and control of antioxidant response, in accordance with our findings (De Sousa et al. 2004). Disruptions in redox homeostasis in these regions, characterized by excessive ROS production and impaired antioxidant defenses, may alter neurotransmitter systems, particularly dopaminergic and glutamatergic signaling, which are important for emotional regulation (Avshalumov et al. 2003; Boldyrev et al. 2004; Coyle and Puttfarcken 1993). Furthermore, elevated oxidative damage to lipids, proteins, and nucleic acids has been associated with depressive‐ and anxiety‐like behavioral phenotypes in preclinical models, and the striatum is one region in which stress‐related oxidative imbalance may contribute to these effects (Bouayed et al. 2009; Che et al. 2015; Ng et al. 2008).

Interestingly, the DCM fraction slightly increased ROS levels in the cerebellum and reduced GR in the cortex, changes that were unexpected. Also, reduction of ROS and TBARS in the hippocampus under stress can look counterintuitive. The biological significance of these changes is unclear, but they might be transient or threatened with compensatory feedback mechanisms. Furthermore, the cerebellum differs from the hippocampus in having high mitochondrial density and a distinct antioxidant enzyme baseline, which can yield increased ROS in the cerebellum and reduced ROS in the hippocampus. The reduction in ROS and TBARS in the hippocampus following stress may reflect an adaptive hermetic response characterized by activation of antioxidant pathways (e.g., Nrf2), and enhanced redox buffering capacity. This effect is consistent with early‐phase stress responses rather than chronic oxidative damage. Importantly, these did not appear to translate into any adverse behavioral outcomes or overt toxicity. Overall, these data suggest that *B. forficata* exerts adaptogenic‐like effects by modulating stress‐sensitive redox circuits in a region‐specific manner, with predominant protection in striatal pathways and complementary antioxidant adjustments in hippocampus and cortex.

Finally, we acknowledge the specific scope of the present study. While our analysis provides a robust putative phytochemical profile, future validation with authentic standards is warranted. Our experimental design deliberately focused on an acute stress paradigm to evaluate short‐term, preventive adaptogenic‐like potential. Thus, while positive pharmacological control was omitted, these findings establish a strong foundation for future chronic stress models. The behavioral battery yielded consistent functional outcomes, though standard consecutive assays carry inherent translational constraints; in this context, the forced swim test remains open to interpretive debate, as immobility may reflect coping‐related processes rather than purely behavioral despair (Molendijk and de Kloet 2022). Furthermore, our comprehensive mapping highlighted central redox modulation as a primary mechanism; nevertheless, incorporating specific molecular targets, such as inflammatory cytokines, will be essential to fully elucidate downstream HPA axis regulation. Lastly, as region‐specific biochemical findings were not globally corrected across all independent panels, they serve as a valuable exploration basis for targeted mechanistic investigations.

## 5 Conclusion

In conclusion, this study provides the first evidence of the adaptogenic‐like potential of *B. forficata* leaf extracts (CE and DCM fraction) in a murine model of acute stress. Our findings demonstrate that these extracts effectively mitigate stress‐induced anxiety and depressive‐like behaviors, likely through the modulation of oxidative stress pathways and restoration of antioxidant capacity in key brain regions, such as the striatum and cortex. The presence of bioactive flavonoids, including rutin and quercetin derivatives, supports these neuroprotective effects. Furthermore, the absence of hepatic and renal toxicity highlights the safety profile of these extracts at the tested doses. Collectively, these findings highlight the preclinical preventive strategy and adaptogenic‐like potential of *B. forficata*. However, further studies are required to evaluate its translational therapeutic viability in chronic stress paradigms and clinical settings.

## Author Contributions

**Sofia Alencar Lafetá**: methodology, formal analysis, writing – original draft, investigation, visualization, writing – review and editing. **Thiago Neves Vieira**: investigation, writing – original draft, writing – review and editing, visualization, methodology, formal analysis, data curation, validation. **Rodrigo Rodrigues Franco**: conceptualization, methodology, validation, writing – original draft, writing – review and editing, supervision. **Ana Claudia Gontijo Couto**: methodology, investigation, writing – review and editing. **Vinicius Prado Bittar**: methodology, investigation, writing – review and editing. **Maria Sol Peña Carrillo**: investigation, writing – review and editing. **Renata Roland Teixeira**: supervision, methodology, investigation, writing – review and editing. **André Luis Lopes Saraiva**: conceptualization, methodology, validation, writing – review and editing. **Allisson Benatti Justino**: validation, formal analysis, writing – review and editing, data curation. **Tamara Silva**: writing – review and editing, investigation, methodology. **Veridiana de Melo Rodrigues Ávila**: writing – review and editing, funding acquisition, supervision. **Foued Salmen Espíndola**: writing – review and editing, conceptualization, writing – original draft, resources, supervision, data curation, funding acquisition. **Cássia Regina Silva**: writing – review and editing, conceptualization, writing – original draft, resources, supervision, project administration, data curation, funding acquisition.

## Funding

This study was funded by the Foundation for Research Support of the Minas Gerais State–FAPEMIG (REMITRIBIC, RED‐00031‐21) and the National Institute of Science and Technology in Theranostics and Nanobiotechnology—INCT—TeraNano (National Council for Scientific and Tachnological Development ‐ CNPq grant # 465669/2014–0, http://www.teranano.ufu.br/). F.S.E. grant from CNPq (APQ‐01856‐14) and CNPq/CAPES/FAPEMIG (CNPq‐465669/2014‐0 and FAPEMIG‐CBB‐APQ‐03613‐17). F.S.E. and C.R.S. are grant recipients of CNPq (PQ—Research productivity, process no. 303853/2025‐5 and 311281/2022‐2).

## Ethics Statement

All animal handling and procedures were approved by the Ethics Committee in Animal Experimentation of the Federal University of Uberlândia (CEUA/UFU 012/20).

## Conflicts of Interest

Sofia Alencar Lafetá is employed by Procter and Gamble. The other authors declare no conflicts of interest.

## Supporting information




**Supplementary Table**: brb371819‐sup‐0001‐TableS1.docx

## Data Availability

The data that support the findings of this study are available from the corresponding author upon reasonable request.
