# Supplementary material for: Adaptogenic‐Like Potential of Bauhinia forficata Leaf Extracts: Behavioral and Oxidative Outcomes in an Acute Stress Mouse Model
Source: Brain Behav. 2026 Oct 8;16(10):e71819. doi: 10.1002/brb3.71819 (PMC13647495; doi:10.1002/brb3.71819)
Supplement: Supplementary file 1 [file BRB3-16-e71819-s001.docx]

| **Brain region** | **Oxidative stress biomarkers** | **Unit** | **Naive**  **(± SE)** | **Vehicle**  **(± SE)** | **CE (30mg/kg)**  **(± SE)** | **DCM (10mg/kg)**  **(± SE)** | **One-way ANOVA** |
| --- | --- | --- | --- | --- | --- | --- | --- |
| **Striatum** | SOD | U/mg | 0.79 ±0.14 | **1.37 ±0.2 #**  **p=** **0.0251** | **0.77 ±0.07 ***  **p=** **0.0193** | 1.09 ±0.17 | F(3, 28) = 3.757,  p = 0.0220 |
|  | CAT | U/mg | 1.42 ±0.18 | **2.61±0.55 ##**  **p=** **0.0093** | **1.52±0.06 ***  **p=** **0.0182** | 1.82±0.13 | F(3, 28) = 4.203,  p = 0.0142 |
|  | GPx | U/mg | 20.82 ±1.59 | **29.22 ±3.50 ##**  **p=** **0.0077** | **19.80 ±0.43 ****  **p=** **0.0028** | 23.09±1.37 | F(3, 28) = 5.427,  p = 0.0045 |
|  | GR | U/mg | 5.68 ±0.49 | **8.93 ±1.25 #**  **p=** **0.0102** | **5.99 ±0.20 ***  **p=** **0.0216** | 7.34 ±0.57 | F(3, 28) = 4.156,  p = 0.0148 |
|  | GSH | nmol/mg | 1.25 ±0.12 | **2.34 ±0.34 ###**  **p=** **0.0007** | **1.42 ±0.14 ****  **p=** **0.0039** | 1.81 ±0.15 | F(3, 28) = 6.979,  p = 0.0012 |
|  | FRAP | μmol trolox eq/mg | 41.21 ±5.25 | 44.01 ±5.48 | **70.93 ±12.31 ***  **p=** **0.0376** | **101.48 ±3.19 ******  **p=** **<0,0001** | F(3, 28) = 14.88,  p < 0.0001 |
|  | Sulfhydryl | nmol/mg | 45.51 ±4.10 | 46.75 ±8.85 | 44.11 ±5.11 | 42.66 ±5.38 | F(3, 28) = 0.083,  p = 0.9687 |
|  | ROS | Unit of fluorescence | 986.12 ± 94.86 | **1414.31 ±169.42 #**  **p=** **0.0286** | **963.76 ±32.18 ***  **p=** **0.0205** | 1077.62 ±101.97 | F(3, 28) = 3.538,  p = 0.0273 |
|  | TBARS | nmol MDA/mg | 1.05 ±0.085 | **1.71 ±0.30 ##**  **p=** **0.0052** | **1.09 ±0.04 ****  **p=** **0.0080** | **1.17 ±0.10 ***  **p=** **0.0207** | F(3, 28) = 5.195,  p = 0.0056 |
| **Hippocampus** | SOD | U/mg | 12.39 ±0.89 | 11.47 ±1.06 | 11.97 ±0.79 | 12.69 ±1.02 | F(3, 28) = 0.3263,  p = 0.8063 |
|  | CAT | U/mg | 50.7 ±4.84 | **35.59±2.52##**  **p=** **0.0015** | 31.31±1.96 | 32.65±2.49 | F(3, 28) = 10.71,  p < 0.0001 |
|  | GPx | U/mg | 85.97 ±3.81 | 85.52 ±5.72 | 89.33 ±5.33 | **126.84 ±6.37*****  **p=** **<0.0001** | F(3, 28) = 14.73,  p < 0.0001 |
|  | GR | U/mg | 40.14 ±1.59 | **30.50 ±2.20##**  **p=** **0.0032** | 34.97 ±1.99 | **41.37 ±2.38*****  **p=** **0.0010** | F(3, 28) = 7.072,  p = 0.0011 |
|  | GSH | nmol/mg | 5.37 ±0.7 | 4.47 ±0.41 | 5.22 ±0.64 | **7.38 ±1.08***  **p=** | F(3, 28) = 2.133,  p = 0.1185 |
|  | FRAP | μmol trolox eq/mg | 7.43 ±0.92 | 7.63 ±1.25 | 7.02 ±1.29 | 6.99 ±1.01 | F(3, 28) = 0.077,  p = 0.9716 |
|  | Sulfhydryl | nmol/mg | 176.51 ±4.65 | **132.36 ±5.59##**  **p=** **0.0054** | 153.81 ±8.61 | **178.63 ±17.64****  **p=** **0.0036** | F(3, 28) = 5.673,  p = 0.0036 |
|  | ROS | Unit of fluorescence | 3833.32 ±230.98 | **2787.91 ±157.91##**  **p=** **0.0030** | 3015.81 ±167.48 | 3474.76 ±255.89 | F(3, 28) = 5.307,  p = 0.0050 |
|  | TBARS | nmol MDA/mg | 6.25 ±0.30 | **4.96 ±0.40#**  **p=** **0.0136** | 4.77 ±0.32 | **3.89 ±0.16***  **p=** **0.0471** | F(3, 28) = 10.51,  p < 0.0001 |
| **Cerebellum** | SOD | U/mg | 2.09 ±0.16 | 1.75 ±0.16 | 1.94 ±0.2 | 2.22 ±0.21 | F(3, 28) = 1.197,  p = 0.3290 |
|  | CAT | U/mg | 23.9 ±3.67 | 25.04 ±3.22 | **44.83 ±7.3***  **p=** **0.0159** | 38.08 ±3.33 | F(3, 28) = 4.719,  p = 0.0087 |
|  | GPx | U/mg | 10.55 ±0.68 | 11.77 ±0.91 | 13.04 ±1.53 | 10.28 ±0.62 | F(3, 27) = 1.514,  p = 0.2333 |
|  | GR | U/mg | 17.92 ±0.74 | 27.86 ±4.32 | 30.62 ±3.73 | 18.22 ±1.99 | F(3, 28) = 4.625,  p = 0.0095 |
|  | GSH | nmol/mg | 4.21 ±0.58 | 3.70 ±0.47 | 5.57 ±.65 | **7.06 ±0.88****  **p=** **0.0034** | F(3, 28) = 5.218,  p = 0.0055 |
|  | FRAP | μmol trolox eq/mg | 17.25 ±4.98 | 23.94 ±2.36 | 26.32 ±3.48 | 27.23 ±2.69 | F(3, 28) = 1.636,  p = 0.2036 |
|  | Sulfhydryl | nmol/mg | 47.78 ±14.79 | 32.70 ±2.53 | 45.32 ±5.67 | **66.61 ±9.71***  **p= 0.0342** | F(3, 28) = 2.400,  p = 0.0890 |
|  | ROS | Unit of fluorescence | 1355.45 ±164.38 | 1257.11 ±126.40 | 1573.92 ±128.93 | **2551.5 ±305.66*****  **p= 0.0002** | F(3, 28) = 9.190,  p = 0.0002 |
|  | TBARS | nmol MDA/mg | 69.68 ±4.05 | 66.37 ±4.85 | 71.23 ±1.36 | 67.44 ±3.33 | F(3, 28) = 0.362,  p = 0.7804 |
| **Cortex** | SOD | U/mg | 9.92 ±1.34 | **2.32 ±0.27#**  **p= 0.0127** | **14.61 ±2.25******  **p= <0.0001** | **8.95 ±2.3***  **p=0.0320** | F(3, 28) = 8.394,  p = 0.0004 |
|  | CAT | U/mg | 63.68 ±10.77 | **13.42 ±1.93###**  **p= 0.0005** | **58.34 ±10.55****  **p= 0.0017** | 40.07 ±6.16 | F(3, 28) = 7.651,  p = 0.0007 |
|  | GPx | U/mg | 47.54 ±8.37 | 42.20 ±10.14 | 45.56 ±10.87 | 28.91 ±5.13 | F(3, 28) = 0.939,  p = 0.4348 |
|  | GR | U/mg | 53.44±9.45 | 56.83 ±15.25 | 36.62 ±7.85 | **21.85 ±4.3***  **p= 0.0100** | F(3, 28) = 4.308,  p = 0.0128 |
|  | GSH | nmol/mg | 11.82 ±1.53 | 7.77 ±2.22 | 11.97 ±3.61 | 7.69 ±0.36 | F(3, 28) = 1.559,  p = 0.2214 |
|  | FRAP | μmol trolox eq/mg | 65.27 ±4.36 | 65.81 ±5.54 | 81.66 ±6.68 | 70.65 ±4.41 | F(3, 28) = 2.032,  p = 0.1321 |
|  | Sulfhydryl | nmol/mg | 198.60 ±24.11 | **127.98 ±8.74##**  **p= 0.0083** | 154.43 ±14.0 | 158.34 ±9.57 | F(3, 28) = 3.595,  p = 0.0258 |
|  | ROS | Unit of fluorescence | 14108.23 ±2611.13 | 15960.91 ±4498.48 | 15310.85 ±4293.16 | 15639.5 ±2543.41 | F(3, 28) = 0.055,  p = 0.9825 |
|  | TBARS | nmol MDA/mg | 3.83 ±0.88 | 3.19 ±0.79 | 3.61 ±0.62 | 3.51 ±0.64 | F(3, 28) = 0.128,  p = 0.9423 |

**Table S1. Effects of the crude extract (CE) and dichloromethane (DCM) fraction of Bauhinia forficata on oxidative stress biomarker levels across different brain regions.** Oxidative stress biomarkers evaluated: Superoxide dismutase (SOD), Catalase (CAT), Glutathione Peroxidase (GPx), Glutathione reductase (GR), Glutathione (GSH), Total antioxidant capacity (FRAP), Sulfhydryl, Reactive Oxygen Species (ROS), and Lipid peroxidation (TBARS). Brain regions analyzed: Striatum, Hippocampus, Cerebellum, and Cortex. Groups (n = 8 animals/group): naive, stressed animals treated with vehicle (vehicle group), stressed animals treated with 30 mg/kg of crude extract (CE 30 mg/kg), and stressed animals treated with 10 mg/kg of dichloromethane fraction (DCM 10 mg/kg). Dunnett-adjusted p-values for significant comparisons are displayed below the respective mean ± SEM. Symbols denote the significance thresholds: #p<0.05, ##p<0.01, and ###p<0.001 indicate significant differences between the vehicle group and the naive group; *p<0.05, p<0.01, and ***p<0.001 indicate significant differences between the extract-treated groups and the vehicle group. Data are expressed as mean ± SEM. Statistical analysis was conducted using one-way ANOVA (exact F statistics, degrees of freedom, and p-values are provided in the respective column) followed by Dunnett's post-test.
